# Regulation of breast cancer cell motility by T-cell lymphoma invasion and metastasis-inducing protein

**DOI:** 10.1186/bcr2637

**Published:** 2010-09-06

**Authors:** Homer C Adams, Ru Chen, Zhuoming Liu, Ian P Whitehead

**Affiliations:** 1 Department of Microbiology and Molecular Genetics and the New Jersey Medical School, University Hospital Cancer Center, University of Medicine and Dentistry of New Jersey, 65 Bergen Street, Newark, NJ 07101-1709, USA

## Abstract

**Introduction:**

T-cell lymphoma invasion and metastasis-inducing protein (Tiam1) is an Ras-related C3 botulinum toxin substrate (Rac)-specific guanine nucleotide exchange factor that was isolated based on its ability to induce a metastatic phenotype. In polarized migrating keratinocytes, Tiam1 is found at the leading edge, where it cooperates with the protease-activated receptor 1 (Par1) complex to establish front-to-rear polarity. Although a positive correlation has been observed between Tiam1 expression and tumor grade in a variety of human malignancies, including breast, its role in breast cancer cells has not yet been examined.

**Methods:**

Tiam1 expression and Rac activity were examined in a panel of human breast cancer cell lines that exhibit different degrees of cell motility. The contribution of Tiam1 to cell motility was directly examined by using transwell motility and wound-healing assays.

**Results:**

Although we observed a striking, positive correlation between Tiam1 expression and cell motility in the panel of breast cancer cell lines, we did not observe a correlation between Tiam1 expression and overall levels of Rac activity. Consistent with this, small interfering ribonucleic acid (siRNA)-mediated suppression of Tiam1 expression limits the motility of cell lines in which Tiam1 expression is high (MDA-MB-231 and MDA-MB-453) but does not substantially alter the overall levels of activated Rac. Tiam1 overexpression is also not sufficient to increase the motility of more poorly motile cells (T-47D) or to increase Rac activity. Immunofluorescence and cellular fractionations indicate that Tiam1 is found predominantly in the Golgi of breast cancer cells, and in the latter case, Tiam1 was shown to co-fractionate with a limited pool of Rac1. Consistent with this Golgi localization, Tiam1 supports cell motility and Golgi reorientation in response to serum in a wound-healing assay using MDA-MB-231 and MDA-MB-435S cells.

**Conclusions:**

Tiam1 expression correlates with cell motility in human breast cancer cells and is required to support the motile phenotype. Localization of endogenous Tiam1 to the Golgi, and its demonstrated role in Golgi reorientation, suggest that it may support motility through a mechanism that is discrete from its known function in leading-edge dynamics.

## Introduction

Directed cell migration is a regulated process that occurs during embryonic development [1], wound healing [2], bone remodeling [3], angiogenesis [4], and the immune response [5], and is observed under pathologic conditions such as atherosclerosis [6], tumor cell motility, and metastasis [7]. Features of the polarized, motile cell include the formation of filopodia and lamellipodia at the leading edge, stabilization and localized capture of microtubules at the leading edge, and reorientation of the microtubule organizing center and Golgi complex toward the direction of migration [8]. The reorientation of the Golgi and microtubules may facilitate the trafficking of the protein and lipid components that would be required at the leading edge for membrane remodeling and protrusion to occur [9,10]. Although the cellular activities that are required to support motility were first identified and described in noncancer cells [11], subsequent studies have established that cancer cells, for the most part, use the equivalent molecular mechanisms [12,13].

Members of the Rho family of small GTPases have emerged as key regulators of cell polarity and motility [14,15]. Rho proteins function as binary switches cycling between an active GTP-bound state and an inactive GDP-bound state. The steady-state level of Rho-GTP in cells is determined by the actions of three families of regulatory molecules. Guanine nucleotide exchange factors (GEFs) activate RhoGTPases by catalyzing the exchange of GDP for GTP [16]; RhoGTPase activating proteins inhibit Rho function by stimulating the intrinsic rate of GTP hydrolysis [17]; whereas guanine nucleotide dissociation inhibitors sequester Rho-GDP in an inactive state [18]. Binding to GTP induces a conformational change in a Rho protein, which allows binding and activation of downstream signaling molecules [19]. The Rho family members RhoA, Rac1, and Cdc42 have been identified as key regulators of cell motility, initially based on their ability to regulate the actin cytoskeleton [9]. Subsequent studies revealed an important role for these three Rho family members in virtually all aspects of the motile phenotype, including actin reorganization, microtubule stabilization and capture, centrosome reorientation, and vesicle trafficking from the Golgi [8].

Tiam1 is a Rac-specific GEF [20] that was originally identified in a screen for proteins whose overexpression can cause an invasive phenotype in T-lymphoma cells [21]. Subsequent studies have identified roles for Tiam1 in the regulation of apical-basal polarity in epithelial cells, neurite growth and differentiation, and cell motility [22,23]. Each of these activities involves a regulatory interaction between Tiam1 and the Par polarity complex [22]. Thus, in polarized migrating keratinocytes, Tiam1 associates with Par3 and Protein kinase C-ζ at the leading edge, where it cooperates with the Par complex to establish front-to-rear polarity by promoting the stabilization of the microtubule network [24]. Although these results appear to be in conflict with earlier studies indicating that Tiam1 promotes cell adhesion in contacting keratinocytes [25], it has been proposed that when cell-to-cell contacts are present, the function of Tiam1 is to support apical-basal polarity, but when such contacts are absent, such as in migrating keratinocytes, Tiam1 promotes cell motility by stabilizing front-to-rear polarity [24].

Although most evidence supports a role for Tiam1 in cancer progression, it appears to have both growth-suppressive and growth-promoting functions, depending on the tumor type examined. Like many Rho-specific GEF family members, a derivative of Tiam1 that lacks the amino terminus has oncogenic activity when overexpressed in NIH 3T3 cells [26,27]. Consistent with this oncogenic activity, an examination of 40 human tumor cell lines including neuroblastomas, melanomas, hematopoietic tumors, lung carcinomas, colon carcinomas, and breast carcinomas revealed that Tiam1 was expressed in virtually all the cell lines [28]. A correlation between Tiam1 expression and tumor grade has been observed in breast carcinoma [29], nasopharyngeal carcinoma [30], hepatocellular carcinoma [31], retinoblastoma [32], and prostate cancer [33]. In tumor types in which elevated expression is observed, Tiam1 may support cancer progression by regulating motility and metastasis. The metastatic potential of eight colon tumor cell lines positively correlates with Tiam1 expression [34], and overexpression of Tiam1 contributes to the metastatic potential of colon cancer cells [35,36]. Tiam1 protein expression also correlates with migration capacity in breast tumor cell lines [23], and a close correlation has been observed between increased Tiam1 expression and tumor grade [29]. In mice, Tiam1 expression has been shown to be necessary for Neu-induced, but not Myc-induced, mammary tumor formation [37].

Not all studies support a role for Tiam1 as an oncogene. Thus, in renal cell carcinoma [38], breast carcinoma [39], and gastric cancer [40], an inverse correlation has also been observed between Tiam1 expression and invasive potential, and Tiam1 expression in renal carcinoma cells inhibits migration by promoting E-cadherin-mediated adhesion [38]. Studies using mouse models suggest that Tiam1 may function as both an oncogene and tumor suppressor during tumor progression. Thus, Tiam1-null mice are resistant to Ras- and adenomatous polyposis coli (APC)-induced tumors, but the tumors that do form are more likely to progress [41,42]. Evidence suggests that Tiam1 supports Ras-induced tumorigenesis by inhibiting apoptosis and promoting proliferation, but that the tumors that do grow progress more rapidly because of the normal role of Tiam1 in maintaining cell-to-cell interactions [42]. A similar dual contribution by Tiam1 to tumor progression has been proposed for APC-induced tumors [41]. Collectively, these observations suggest that the contribution of Tiam1 to tumorigenesis is dependent on the tumor type, the degree of tumor progression, and the signaling context. Thus, during early stages of tumor progression, suppression of Tiam1 expression may be necessary to disrupt cell-to-cell adhesions and support epithelial-mesenchymal transition [43]. However, as a limited population of tumor cells acquire an invasive phenotype, Tiam1 expression may be restored to support invasion or polarized motility.

Although it was previously reported that Tiam1 expression is elevated in human breast cancer cell lines [23], the activity of Tiam1 in these cells is unknown. In the current study, we observed a close correlation between Tiam1 expression and motility in a panel of human breast tumor cell lines. Tiam1 expression is required to support cell movement in the more-motile cell lines, but is not sufficient to induce motility in a less-invasive cell line. Both Tiam1 and Rac1 localize to the Golgi apparatus in the more-motile cell lines, and both are required to support serum-induced Golgi reorientation in a wound-healing assay. These results establish a role for Tiam1 in supporting breast tumor cell motility and suggest that this role may be independent of its contributions to leading-edge dynamics.

## Materials and methods

### Molecular constructs

pCDNA3-Tiam1 and pCDNA3-Rac61L(HA) encode full-length human Tiam1 and a hemagglutinin (HA)-epitope tagged, constitutively activated mutant of Rac1, respectively.

### Cell culture and transfection

All cell lines used for this analysis were obtained from American Type Culture Collection. The MDA-MB-231, MDA-MB-435 S, MDA-MB-453, and ZR-75-1 cell lines were cultured in Dulbecco's Modified Eagle Medium (DMEM) supplemented with 10% Fetal Bovine Serum (FBS; Gemini Bio-Products), 100 U/ml penicillin/streptomycin (Cellgro MediaTech), 2 m*M *L-glutamine (Cellgro MediaTech), and 1 m*M *sodium pyruvate (Cellgro MediaTech). T-47 D cells were cultured in Roswell Park Memorial Institute 1640 Medium supplemented with 10% FBS, 100 U/ml penicillin/streptomycin, and 2 m*M *L-glutamine. MCF-10A cells were cultured in 50% DMEM/50% Hams F12 Medium supplemented with 5% horse serum (Sigma-Aldrich), 0.5 mg/ml hydrocortisone (Sigma-Aldrich), 10 μg/ml human epidermal growth factor (Sigma-Aldrich),100 ng/ml Cholera toxin (Sigma-Aldrich), 5 mg/ml insulin (Invitrogen), and 50 U/ml penicillin/streptomycin. To establish stable cell lines, cells were transfected with Lipofectamine 2000 reagent (Invitrogen) according to the manufacturer's recommended protocol and then selected in growth medium supplemented with Geneticin (400 ng/ml, Invitrogen). For each transfection, more than 200 drug-resistant colonies were combined to generate a polyclonal stable cell line, and three independent cell lines were generated and tested for each construct.

### Reagents

Brefeldin A (BFA: 10 μg/ml; Invitrogen) was added to cells in serum-starved media (0.5% serum) for 30 min, and then cells were allowed a 3-hour recovery period before staining or transwell motility assays. The Rac1 inhibitor (50 μ*M *NSC23766; Calbiochem) was added under serum-starved conditions for 12 hours and then was maintained through the course of the Golgi-reorientation assays. Antibodies used for Western blots and immunofluorescence included anti-Tiam1 (Santa Cruz, sc-872), anti-Vav (Santa Cruz, sc-132), anti-Cdc42 (BD Transduction Laboratories), anti-Rac1 (BD Transduction Laboratories, and Santa Cruz, sc-95), anti-α tubulin (Sigma-Aldrich), anti-α actinin (Sigma-Aldrich), anti-HA (BAbCO), anti-GM130 (BD Transduction Laboratories), and anti-Rho A (Santa Cruz, sc-418). Western blot analysis was performed as previously described [44]. A blocking peptide for the Tiam1 antibody (Santa Cruz, sc872P) was used to validate the specificity of the antibody according to the manufacturer's protocols.

### Cdc42 and Rac1 activation assays

Affinity purification assays to measure the levels of endogenous GTP-bound Rac1 and Cdc42 were performed by using the Cdc42- and Rac-binding domain of p21 activated kinase (GST-PAK), as previously described [45].

### Transfection of siRNA

siRNAs specific for Tiam1 or controls (Santa Cruz) were transiently transfected into cells by using SiLentFectTM lipid reagent (Bio-Rad), according to the manufacturer's protocols. The minimal concentration of siRNAs that could consistently reduce total Tiam1 protein expression by greater than 75% was determined with Western blot analysis. The viability of the transfected cells was confirmed with the trypan blue exclusion method.

### Motility assays

Transwell motility assays were performed as previously described [46]. Subconfluent cells were detached with 0.05% trypsin, and 2.5 × 10^4 ^cells were loaded into the top chamber of transwells coated from the underside with collagen I, and then allowed to migrate. Cells that migrated to the underside of the membranes were fixed and stained with Diff-Quik (Dade Behring) and counted. All experiments were performed in triplicate, and eight to 12 fields of view for each transwell were counted. The average number of cells per field was then calculated. For wound-healing assays, confluent cells were serum starved overnight (0.5% serum). The following day, cells were wounded with a sterile 200-ml pipette tip and then cultured in complete media (10% serum). Designated points on the wound were selected randomly and photographed at indicated intervals to observe differences in wound closure. All experiments were performed in triplicate. Where indicated, cells were transiently transfected with siRNAs 48 hours before serum starvation.

### Golgi orientation assays

Cells were plated on poly-L lysine (0.01%; Sigma-Aldrich)-coated coverslips and allowed to reach >80% confluency. Cells were then transfected with siRNAs for 24 hours and then grown to ≥95% confluency. Cells were then serum starved (0.5% serum) for 12 hours, wounded with a sterile 200-ml pipette tip, and then cultured in complete media (10% serum) for the duration of the experiment. For cells treated with NSC23766, the inhibitor was added during serum starvation and maintained throughout the experiment. Cells were then fixed with 3.7% paraformaldehyde (Sigma-Aldrich) for 20 min and stained for phalloidin, GM130, and 4',6-diamidino-2-phenylindole (DAPI). Cells were scored as wound oriented if the majority of the Golgi apparatus was localized in the third of the cell that faced the wound. Images were obtained by using a Zeiss Axio 40 CFL with Observer Z1 inverted microscopy and AxioVision software (Carl Zeiss). All experiments were performed in triplicate.

### Immunofluorescence

Cells were plated onto poly-L-lysine-coated coverslips. They were then fixed with 3.7% paraformaldehyde for 20 min, washed with 1× phosphate-buffered saline (PBS), and then blocked in 1× PBS containing 3% bovine serum albumin (Equitech-Bio) and 0.1% Triton X-100 (J.T. Baker) for 1 hour. After washing with 1× PBS, cells were incubated with primary antibodies for 1 hour, washed with PBS, and then incubated for 30 min with secondary antibodies. Where indicated, phalloidin staining was performed with either Alexa Fluor 488 or 546 (Invitrogen) at a dilution of 1:50 for 20 min. Cells were viewed with a ZEISS Axiovert 200 M microscope fitted with an ApoTome Imaging system. Image stacks in the axial direction were acquired, and all images shown are from a representative axial plane.

### Cellular fractionation

Cell samples were separated out into molar density fractions as described by others [47]. Interfaces between gradient molarities were obtained and mixed with 2× SDS loading buffer, boiled, and centrifuged at high speed before separation of proteins on SDS polyacrylamide gels for Western blot analysis.

### Statistics

The *P *values were determined by using a Student's *t *test for nonpaired values. Values are typically given as mean ± SEM.

## Results

### Tiam1 is differentially expressed in human breast epithelial cell lines

**Figure 1:**
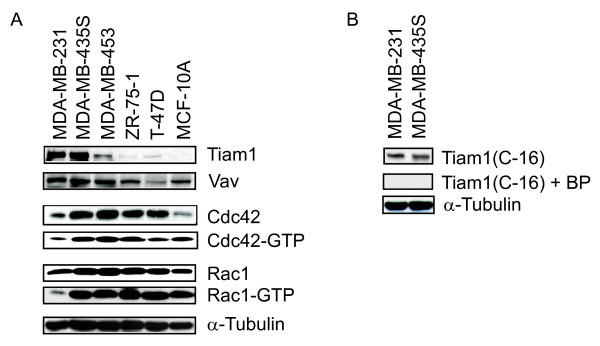
**Tiam1 expression correlates with motility, but not Rac activation, in human breast cancer cell lines**. **(a) **Lysates were collected from a panel of human breast cancer cell lines and examined with Western blot for expression of Tiam1, Vav, Rac1, Cdc42, and α-tubulin (loading control). Lysates were also examined with affinity precipitation assays for levels of activated Rac1 (Rac1-GTP) and Cdc42 (Cdc42-GTP). Cell lines are ordered from highest motility on the left to lowest motility on the right. **(b) **The indicated cell lines were examined with Western blot by using a Tiam1 antibody (c-16) in the presence (upper panel) or absence (second panel) of blocking peptide, as described in Materials and Methods. All blots shown are representative of at least three independent experiments.

To determine whether Tiam1 is expressed in tumor-derived human breast epithelial cells, lysates were collected from a panel of cell lines and examined with Western blot with an antibody specific for Tiam1 (Figure 1). The panel consisted of five tumor cell lines with different degrees of motility (MDA-MB-231, MDA-MB-435 S, MDA-MB-453, ZR-75-1, and T-47D), and a nonmotile, nontransformed human breast epithelial cell line (MCF-10A) (Figure S1 in Additional File 1). The Tiam1 antibody detects a band at approximately 200 kDa, which is differentially expressed among panel members (Figure 1a). This band corresponds closely with the predicted size of full-length Tiam1 and is not detected when the antibody is used in the presence of a blocking peptide (Figure 1b). The level of expression of Tiam1 in this panel is positively correlated with cell motility. Thus, high expression is observed in the MDA-MB-231 and MDA-MB-435S cells, modest expression in the MDA-MB-453 cells, low expression in ZR-75-1 and T-47 D cells, and no expression is observed in the MCF-10A cells. For comparison, we also examined the expression of Vav, a second GEF that predominantly uses Rac as a substrate. The Vav antibody detects a predominant band at 130 kDa, which corresponds closely with the predicted molecular mass of the protein. Unlike Tiam1, Vav expression is detected at roughly equivalent levels in all cell lines except T-47 D, in which the expression is reproducibly low. To demonstrate that differences in levels of expression could not be attributed to uneven loading, the blot was then stripped and reprobed with an antibody specific for α-tubulin.

### Tiam1 expression does not correlate with total Rac expression and activity

It was previously demonstrated that Tiam1 can activate Rac1 and Cdc42 in *in vitro *exchange assays, and Rac1 in cell-based assays [20]. To determine whether the elevated levels of Tiam1 that we observed in the more-motile cell lines is associated with increased levels of activated Rac1, GST-based affinity precipitation assays were used to determine the relative levels of GTP-Rac. For comparison, levels of activated Cdc42 were also measured. Initially lysates were collected from the cell lines and examined for expression of total Rac1 and Cdc42 (Figure 1a). Among panel members, no correlation was observed between total Rac expression and the expression of Tiam1 or Vav. All cell lines express both Rac and Cdc42, with the lowest levels in the highly motile MDA-MB-231 cells and the non-motile MCF-10A cells. Next, we affinity precipitated GTP•Rac and GTP•Cdc42 from the cell lysates by using bacterially purified GST-Pak. Surprisingly, no correlation was found between the levels of activated Rac and Cdc42 and the motility of these cell lines. Thus, all members of the panel have roughly equivalent levels of activated Rac, except the highly motile MDA-MB-231 cells, which have very low levels. A similar pattern is observed for Cdc42, except that activated Cdc42 is also low in the T-47 D cells. We conclude from this analysis that (a) high expression levels of a Rac-specific exchange factor does not necessarily correlate with high overall Rac activation, and (b) overall levels of activated Rac and Cdc42 do not necessarily correlate with cell motility in breast tumor cell lines.

### Tiam1 expression is necessary but not sufficient for motility in breast tumor cells

**Figure 2:**
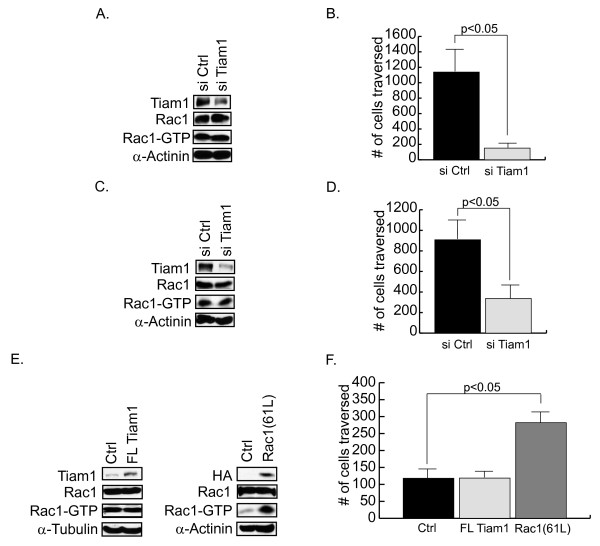
**Tiam1 supports motility in human breast cancer cell lines**. MDA-MB-231 **(a, b) **and MDA-MB-453 **(c, d) **cells were transfected with either control siRNAs (si Ctrl) or siRNAs targeting Tiam1 (si Tiam1). (a, c) Lysates were collected at 48 hours and examined with Western blot for expression of Tiam1, Rac1, and α-actinin (loading control), and with affinity precipitation assays for levels of activated Rac1 (Rac1-GTP). (b, d) Cells from replicate plates were also examined in transwell motility assays as described in Materials and Methods. Cell migration was quantified at either 6 hours (MDA-MB-231) or 48 hours (MDA-MB-453). **(e) **T47 D cell lines were established that stably express full-length (FL) Tiam1, an HA-epitope tagged, activated derivative of Rac1 (Rac1(61L)), or cognate vector (Ctrl), as described in Materials and Methods. Lysates were collected and examined with Western blot for expression of total Rac1, Rac1(61L) (HA), and Tiam1, and with affinity precipitation assay for levels of activated Rac1 (Rac1-GTP). The expression of α-tubulin or α-actinin was also measured as a loading control, as indicated. **(f) **Cells also were examined in transwell motility assays. Cell migration was quantified at 24 hours. Data shown are representative of three independently isolated stable cell lines examined in triplicate experiments (BP).

Because we observed a positive correlation between motility and Tiam1 expression in our panel of tumor cell lines, we determined whether Tiam1 supports the motile phenotype. For this analysis, we used siRNAs targeted at Tiam1 in the MDA-MB-231 and MDA-MB-453 cell lines, both of which express substantial levels of Tiam1. Recent studies have drawn into question the origin of the MDA-MB-435S cell line [48,49], so it was not initially included in this analysis. In the MDA-MB-231 and MDA-MB-453 cell lines, we are able consistently to suppress the expression of Tiam1 by 80% (Figure 2a) and 90% (Figure 2c), respectively, and this is associated with a corresponding reduction in motility in transwell assays (Figure 2b and 2d). Specific targeting of the 200-kDa band by the Tiam1-specific siRNAs further confirmed the identity of the endogenous protein. Surprisingly, a reduction in Tiam1 expression is not associated with a significant decrease in overall levels of activated Rac in either cell line (Figure 2a and 2c). This suggests that either Tiam1 is regulating only a small fraction of total Rac activity in these cells, or it is regulating motility in a Rac-independent manner.

Although our results clearly indicate that Tiam1 is required for breast cancer cell motility, we also wished to determine whether it was sufficient to induce a motile phenotype. For this analysis, we stably expressed full-length Tiam1, or an activated derivative of Rac1 (Rac1(61L)), in T-47 D cells (Figure 2e). We chose this cell line because it expresses low levels of endogenous Tiam1 (Figure 1), and we had previously shown that overexpression of activated Rac is sufficient to induce a motile phenotype [50]. Consistent with our previous results, activated Rac induces a threefold increase in the motility of these cells in transwell assays (Figure 2f). In contrast, full-length Tiam1 does not increase endogenous levels of activated Rac (Figure 2e) or the motility (Figure 2f) of these cells. Overexpression of Tiam1 in a more-motile cell line (MDA-MB-435S) (Figure S2 in Additional file 2) did increase endogenous levels of activated Rac, thus confirming the integrity of the construct. We conclude that although Tiam1 supports cell movement in highly motile cell lines, overexpression of full-length Tiam1 is not sufficient to induce cell movement in a poorly motile cell line.

### Tiam1 colocalizes with Golgi markers in breast tumor cells

**Figure 3:**
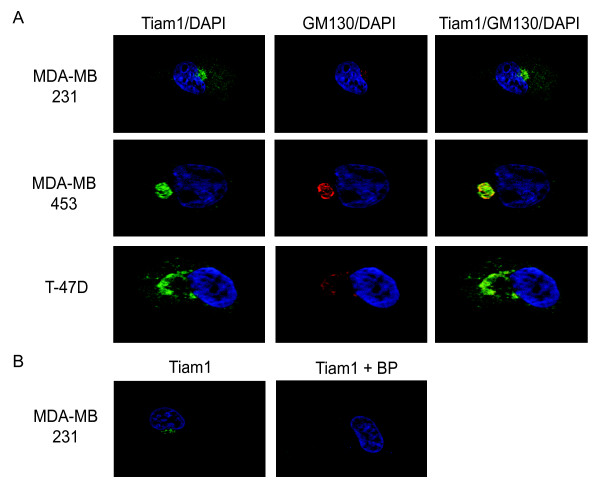
**Endogenous Tiam1 localizes to the Golgi apparatus in breast cancer cells**. **(a) **Cell lines were plated on collagen I (50 μg/ml)-coated coverslips overnight, and then fixed and stained for Tiam1 (green), GM130 (red; Golgi marker), and DAPI (blue). **(b) **Localization of endogenous Tiam1 in MDA-MB-231 cells also was examined in the presence or absence of blocking peptide.

Although ectopically expressed Tiam1 is typically found in the cytoplasm [20,24] or at the leading edge of motile cells [24], we determined whether the cellular distribution of endogenous Tiam1 is different in motile breast epithelial cells. For this analysis, we used co-immunofluorescence on the MDA-MB-231, MDA-MB-453, and T47 D cells by using antibodies for Tiam1 and the Golgi marker, GM130 (Figure 3a). Consistent with the overexpression studies, a diffuse cytoplasmic staining of Tiam1 was observed in all three cell lines. However, strong localized staining also was observed in the perinuclear region, which corresponds precisely with the GM130 marker. No staining was observed at the plasma membrane in any of the three cell types examined. To confirm that the perinuclear staining corresponds to an endogenous pool of Tiam1, we then repeated the immunofluorescence in the MDA-MB-231 cells in the presence, or absence, of the Tiam1 blocking peptide (Figure 3b). In the presence of the blocking peptide, no perinuclear staining was observed in any cells examined.

**Figure 4:**
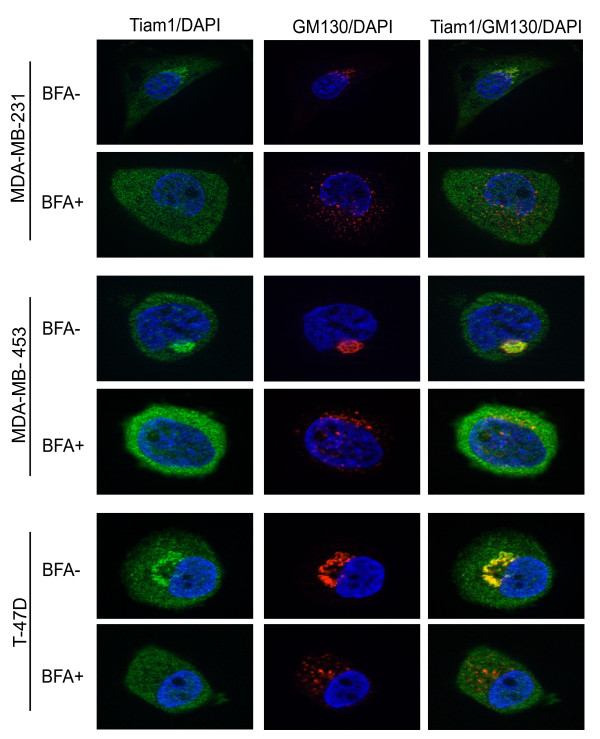
**Brefeldin A disrupts the cellular distribution of Tiam1**. Cells were cultured in the presence (10 μg/ml; BFA^+^) or absence (BFA^-^) of Brefeldin A for 30 minutes under serum-starved conditions. Cells were allowed to recover for an additional 3 hours and were then fixed and stained for Tiam1 (green), GM130 (red), and DAPI (blue).

**Figure 5:**
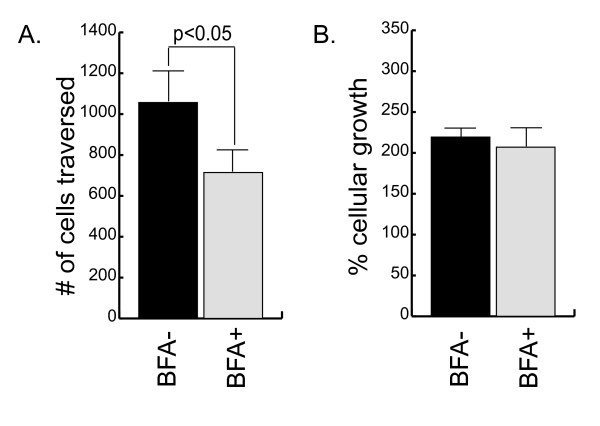
**Golgi-dependent motility in MDA-MB-231 cells**. **(a) **MDA-MB-231 cells were cultured in the presence (10 μg/ml; BFA^+^) or absence (BFA^-^) of Brefeldin A for 30 minutes under serum-starved conditions. Cells were allowed to recover for an additional 3 hours and then examined in transwell motility assays, as described in Methods. Cell motility was quantified at 3 hours. **(b) **In parallel cultures, cells were treated with Brefeldin A as described earlier, and then equal numbers of cells were plated on collagen. The number of cells was counted at 24 hours and expressed as a percentage of cells plated. Data shown are an average of three independent experiments.

To confirm that the Tiam1 and GM130 antibodies are recognizing the Golgi compartment, the co-localization studies were repeated in the presence or absence of BFA (Figure 4). In all three cell lines, treatment with BFA completely disrupted the perinuclear accumulation and co-localization of both GM130 and Tiam1. To determine whether the localization of Tiam1 to the Golgi could account for its contribution to cell motility, MDA-MB-231 cells were then pretreated for 30 minutes with BFA, allowed to recover for 3 hours, and then examined in transwell motility assays. The BFA treatment significantly reduced the motility of the MDA-MB-231 cells, thus implicating the integrity of the Golgi in the movement of these cells (Figure 5a). In parallel cultures, cells were examined for proliferative potential (Figure 5b) and by trypan blue exclusion (not shown) to confirm that the difference in motility could not be attributed to a decrease in cell growth, or an increase in cell death. Because of the longer time required to measure the motility of the MDA-MB-453 cells (24 vs. 3 hours) and the toxicity associated with extended exposure to BFA, an equivalent analysis could not be performed in this cell type.

### Localization of Rac to the Golgi of breast epithelial cells

**Figure 6:**
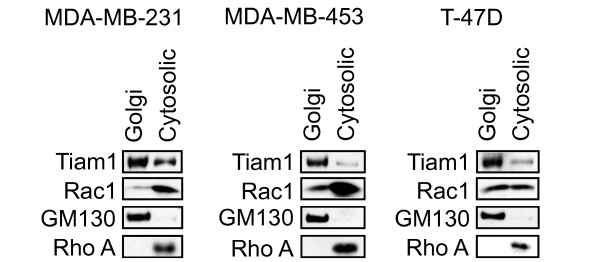
**Tiam1 and Rac1 colocalize to the Golgi apparatus in human breast cancer cell lines**. Golgi and cytosolic fractions were purified from the indicated cell lines, as described in Materials and Methods. Fractions were then examined with Western blot for expression of Tiam1, Rac1, GM130, and RhoA.

In a previous study using live-cell imaging of polarized, motile Swiss 3T3 fibroblasts, a small localized pool of Rac activity was observed in a juxtanuclear region that was consistent with the endoplasmic reticulum or the Golgi or both [51]. Thus, we determined whether Rac is also localized to the Golgi apparatus in MDA-MB-231, MDA-MB-453, and T-47 D cells. Initially, we performed immunofluorescence studies, which failed to provide any definitive evidence for Rac localization to the Golgi in these cell types (Figure S3 in Additional file 3). Because our Rac activation assays suggest that Tiam1 is likely to be regulating only a small pool of Rac, we also performed Golgi fractionations followed by Western blots by using antibodies for Tiam1 and Rac1 (Figure 6). RhoA, which is found almost exclusively in the cytoplasm, was included as a negative control, whereas GM130 was used as a positive control. As expected from our immunofluorescence studies, we observed that a 200-kDa band that is recognized by the Tiam1 antibody is present in both the Golgi compartment and in the cytoplasm in all three cell lines. Similarly, Rac is also detected in both compartments in all three cell lines. The purity of the fractionation was confirmed with the RhoA antibody, which does not cross-react with the Golgi fraction, and the GM130 antibody, which does not cross-react with the cytosolic fraction. These results confirm the presence of both Tiam1 and Rac1 in the Golgi of human breast epithelial cells.

### Tiam1 regulates Golgi reorientation in MDA-MB-231 cells

**Figure 7:**
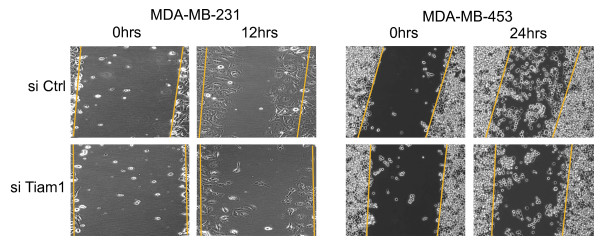
**Tiam1 supports breast cancer cell motility in wound-healing assays**. Plates of confluent MDA-MB-231 and MDA-MB-453 cells were transfected overnight with 200 n*M *control (si Ctrl) or Tiam1 (si Tiam1) siRNA. Cells were then examined in a wound-healing assay, as described in Materials and Methods. Wound closure was measured at 12 hours (MDA-MB-231) or 24 hours (MDA-MB 453). Orange lines indicate the original wound edges. Images are representative of at least 10 independent locations that were monitored over the length of each wound. Orange lines indicate original wound edge.

Directed motility in mammalian cells typically requires repositioning of the Golgi toward the leading edge [8]. This reorientation can be observed in wound-healing assays when serum is added to serum-starved cells. Because Rac1 and Tiam1 are both found in the Golgi of the MDA-MB-231 and MDA-MB-453 cells, we determined whether they regulate Golgi reorientation in these two cell types. Initially we determined whether Tiam1 supports the motility of these cell lines in a wound-healing assay. For this analysis, cells were transfected with control or Tiam1 siRNAs, serum starved overnight, and then wounded with a pipette tip. Cells were then fed 10% serum, and migration into the wound was monitored. Although the MDA-MB-453 cells migrate more slowly into the wound than do the MDA-MB-231 cells, in both cell lines, substantial impairment in cell motility is observed when cells are treated with the Tiam1 siRNAs (Figure 7). This difference in motility is readily apparent at 12 hours with the MDA-MB-231 cells and at 24 hours with the MDA-MB-453 cells.

**Figure 8:**
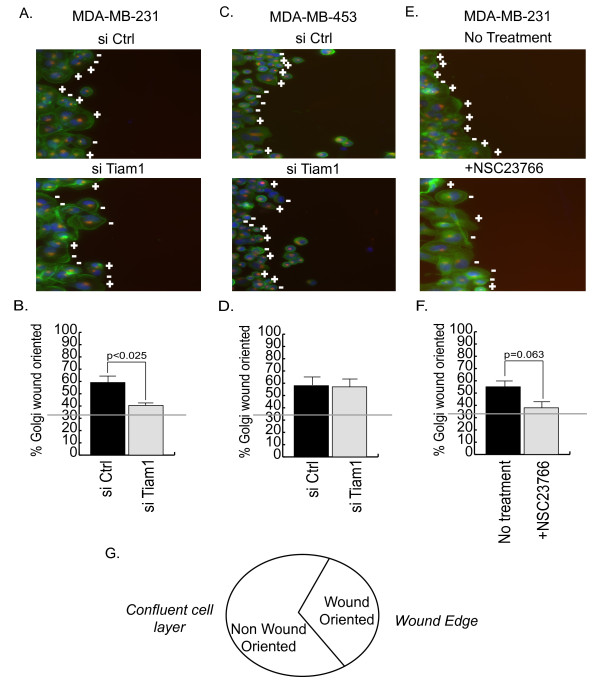
**Golgi reorientation in MDA-MB-231 cells is supported by Tiam1 and Rac1**. MDA-MB-231 **(a, b, e, f) **and MDA-MB-453 **(c, d) **cells were examined for serum-dependent Golgi reorientation in a wound-healing assay, as described in Materials and Methods. (a, c) Confluent cells were transfected with either 200 n*M *control siRNA (si Ctrl) or Tiam1 siRNA (si Tiam1) overnight, serum starved for 12 hours, and then wounded. Alternatively, nontransfected cells were serum starved overnight in the presence or absence of 50 μ*M *Rac1 inhibitor (+NSC23766) and were then wounded (e). After wounding, cells were cultured in 10% serum for 90 minutes (MDA-MB-231) or 210 minutes (MDA-MB-453), and then fixed and stained for phalloidin (green), GM130 (red), and DAPI (blue). Golgi orientation was then scored. Cells treated with the Rac inhibitor were maintained on the inhibitor throughout the course of the experiment. a, c, and e show the results of a typical reorientation experiment, with plus signs indicating cells with Golgi oriented toward the wound, whereas minus signs indicate no orientation. b, d, and f show quantification of three independent experiments. **(g) **Schematic showing the method of scoring of the reorientation experiments.

Next we determined whether impaired motility was associated with a failure to orient the Golgi toward the wound (Figure 8). For this analysis, cells were transfected with control siRNAs or Tiam1 siRNAs and then serum starved overnight. In addition, untransfected cells were serum starved in the presence of the Rac inhibitor. Cells were then wounded, and serum was added for 90 minutes (MDA-MB-231) or 210 min (MDA-MB-453) to induce Golgi re-orientation toward the wound. Cells were then fixed and stained with a Golgi marker. Cells at the wound edge were then divided into three equal sectors, and reorientation of the Golgi into the sector facing the wound was scored (see Figure 8g for schematic). In cells treated with control siRNAs, approximately one third of the cells are orientated toward the Golgi before serum stimulation, which represents the expected random probability (the horizontal lines in Figures 8b, d, and 8f represent this baseline). After serum stimulation, the percentage of control cells in which the Golgi is oriented towards the wound increases to approximately 60% in both cell types (Figure 8b, d, and 8f). In the MDA-MB-231 cells, this reorientation is substantially reduced if Tiam1 expression is suppressed (Figure 8b) or Rac1 is inhibited (Figure 8c). In contrast, Golgi reorientation in the MDA-MB-453 cells appears to be Tiam1 independent (Figure 8d).

**Figure 9:**
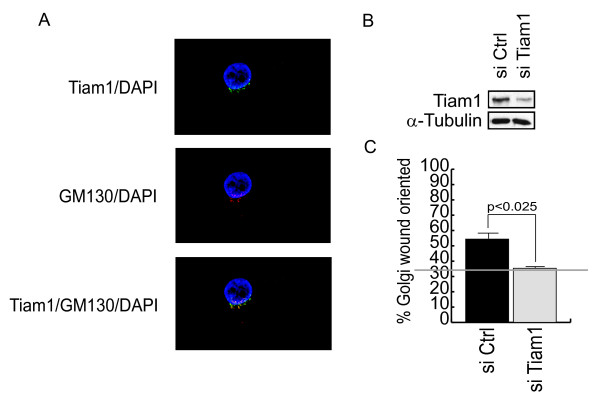
**Golgi reorientation in MDA-MB-435S cells is supported by Tiam1**. **(a) **MDA-MB-435S cells were plated on collagen I (50 μg/ml)-coated coverslips overnight, and then fixed and stained for Tiam1 (green), GM130 (red; Golgi marker), and DAPI (blue). **(b) **Confluent cells were transfected with either 200 n*M *control siRNA (si Ctrl) or Tiam1 siRNA (si Tiam1) overnight and then examined with Western blot for suppression of Tiam1 expression. **(c) **Cells from parallel cultures were then serum starved for 12 hours, wounded, and examined for serum-dependent Golgi reorientation, as described in Materials and Methods. Quantification is shown for three independent experiments.

To determine whether the contribution of Tiam1 to Golgi reorientation is restricted to the MDA-MB-231 cell line, we examined a third tumor cell line that expresses high levels of Tiam1, MDA-MB-435 S. Like all other cell lines we examined, Tiam1 is found in the perinuclear region of these cells, where it co-localizes with a marker for the Golgi (Figure 9a). Similar to the MDA-MB-231 cells, suppression of expression of Tiam1 by siRNA in these cells (Figure 9b) completely eliminates Golgi reorientation in response to serum (Figure 9c).

## Discussion

Although several reports have indicated that Tiam1 is overexpressed in breast tumors, the function of Tiam1 in human, tumor-derived breast epithelial cells has not been determined [23,28]. In this current study, we observed a positive correlation between Tiam1 expression and motility in a panel of breast tumor cell lines. Although Tiam1 has been well characterized as a Rac-specific GEF [20], we do not observe a correlation between Tiam1 expression and overall Rac activity, and reduced expression of Tiam1 does not have an appreciable effect on Rac-GTP levels. This suggests that either Tiam1 is unlinked from Rac in these cell types, or it is regulating only a small fraction of cellular Rac. Consistent with this latter possibility, we observed that endogenous Tiam1 is concentrated in the Golgi complex where it co-localizes with a small fraction of the total cellular Rac. We also observed that at least two other Rac exchange factors, Vav1 (current study) and Dbl (not shown), are widely expressed in our panel, which suggests that total Rac activity in breast tumor cells may be determined by the cumulative activity of multiple RacGEFs. In such a circumstance, the reduced expression of one RacGEF may not have a discernable effect on total cellular levels of activated Rac. Our observation that the suppression of Tiam1 expression can substantially influence cell motility, without affecting total Rac1 levels, suggests that GEFs could be used selectively to target functions of GTPases that support the tumor phenotype, while avoiding targeting of additional functions that may be required to support overall cell viability.

Our results indicate that the high level of Tiam1 expression that we observed in MDA-MB-231 and MDA-MB-453 cells supports the motile phenotype in both transwell and wound-healing assays. However, stable expression of full-length Tiam1 in a poorly motile cell line, such as T-47 D, is not sufficient to induce a motile phenotype. Although activated Rac is sufficient to increase the motility of these cells, Tiam1 does not cause any appreciable increase in Rac-GTP levels when overexpressed. Because Tiam1 has a similar cellular distribution in the highly motile MDA-MB-231, MDA-MB-435 S, and MDA-MB-453 cells and in the poorly motile T-47 D cells, its contribution to cell motility may be dependent on additional activating signals. Consistent with this possibility, we observed elevated levels of activated Rac1 when we transfected the more-motile MDA-MB-435S cell line with Tiam1. Like most RhoGEF family members, Tiam1 contains an amino-terminal autoinhibitory domain, which must be relieved for it to interact with and activate its substrate [52]. Many GEF family members have been identified based on their transforming activity in cDNA expression screens, yet in virtually all cases, the cDNAs are truncated and lack the amino-terminal autoinhibitory domain [53]. Because such activating truncations have not been identified in tumors, the contribution of RhoGEFs such as Tiam1 to tumorigenesis may be dependent on additional activating signals that may be present in some cell types, but not in others. For example, Tiam1 is known to be regulated by direct binding to activated Ras [54]. Whereas T-47 D cells contain wild-type copies of all three Ras genes, MDA-MB-231 cells contain an activating mutation in K-Ras, which in turn may stimulate Tiam1 activity [55]. Similarly, Tiam1 has been shown to be activated in breast epithelial cells by heregulin 2-mediated signaling [29]. Whereas T-47 D cells have normal levels of HER2, MDA-MB-453 cells are HER2-positive [56], which may contribute to Tiam1 activation in this cell type.

Several studies in other cell types have indicated that both endogenous and overexpressed Tiam1 is diffusely localized within the cytoplasm [20,57]. This is considered to be inactive Tiam1 that will redistribute to membrane ruffles in response to various extracellular signals [26,58]. For example, with lysophosphatidic acid and platelet-derived growth factor stimulation of NIH 3T3 cells, serine phosphorylation and membrane translocation of Tiam1 is observed [58]. Although we have also observed that Tiam1 is localized within the cytoplasm in MDA-MB-231, MDA-MB-453, MDA-MB-435 S, and T-47 D cell lines, we did not observe any accumulation of Tiam1 at the plasma membrane, or within lamellipodia or membrane ruffles. This is particularly apparent in the MDA-MB-231 cells, which have a striking mesenchymal morphology with numerous membrane protrusions. However, we observed a strong perinuclear accumulation of Tiam1 in all four cell lines that coincides with a marker for the Golgi, and cellular fractionations confirmed that Tiam1 is associated with this organelle. Treatment of the MDA-MB-231 cells with BFA disrupted the association of Tiam1 with the Golgi and reduced cell motility, suggesting that this association could account for the contribution of Tiam1 to cell motility. Although the BFA treatment had a weaker effect on cell motility than Tiam1 siRNAs, this is likely because the BFA treatments used in our analysis were mild, and of very short duration, to avoid the toxicity normally associated with this compound. To our knowledge, this represents the first demonstration of Golgi localization for endogenous Tiam1 and suggests that it may play a role in cell motility that has not been previously appreciated.

Unlike endogenous Cdc42, which is predominantly localized to the Golgi in mammalian cells [59-61], the localization and activity of Rac in the Golgi is less clear. Antibodies for endogenous Rac typically do not strongly stain the Golgi, and we did not observe such staining in the MDA-MB-231, MDA-MB-453, and T-47 D cells. However, by using live-cell imaging of polarized, motile Swiss 3T3 fibroblasts, Rac activity was observed in a juxtanuclear region that was consistent with the ER or the Golgi [51]. This suggests that the Golgi may contain a small fraction of cellular Rac, which is required to support the motile phenotype. Consistent with this possibility, we used fractionations to confirm that Rac is in the Golgi of MDA-MB-231, MDA-MD-453, and T-47 D cells, and thus may be the target for Tiam1 in this organelle. Because this probably represents a small fraction of the total cellular Rac in these cells, this could account for why we did not observe substantive changes in overall Rac activity when Tiam1 expression was suppressed in these cell lines.

Consistent with a role for Tiam1 in the Golgi, we observed that MDA-MB-231 and MDA-MB-435S cells do not efficiently polarize their Golgi in a wound-healing assay if Tiam1 expression is suppressed or Rac1 is inhibited (MDA-MB-231 cells). Although these cells are still able to migrate into the wound, they do so less efficiently, which likely reflects the lack of polarization. Thus, Tiam1 may be required for polarization, but not cell movement *per se*. The molecular mechanisms that regulate Golgi reorientation during the directed migration of tumor cells is unclear but may involve localized remodeling of the actin cytoskeleton. Both actin and actin-binding proteins have been localized to the Golgi [62], and actin-depolymerizing drugs have been shown to disrupt Golgi morphology and positioning [63-65]. In HEK and COS7 cells, a fraction of Rac has been shown to co-localize with the RacGAP, OCRL1, and γ-adaptin in the trans-Golgi network, which suggests a role for Rac, and possibly Tiam1, in the secretory pathway [66]. In migrating keratinocytes, Tiam1 associates with the Par complex at the leading edge to promote the stabilization of the microtubule network [24]. Although we have not detected Tiam1 at the leading edge of MDA-MB-231 cells, it may function in the Golgi to facilitate reorientation of the microtubule-organizing apparatus and stabilization of microtubules directed toward the leading edge.

Although loss of Tiam1 expression had a profound effect on Golgi reorientation in MDA-MB-231 and MDA-MB-435S cells, it does not appear to support reorientation in MDA-MB-453 cells. This suggests that breast tumor cell lines regulate Golgi reorientation through distinct mechanisms and that Tiam1 can regulate multiple aspects of the motile phenotype. With regard to Golgi reorientation, recent studies in fibroblasts suggest that cell-specific diversity may occur in this process, and that Golgi reorientation may have a different function in slow-moving cells when compared with faster-moving cells [67]. In this regard, it is of note that the MDA-MB-231 and MDA-MB-435S cells are more motile than the MDA-MB-453 cells and have a strikingly different morphology. Thus, the MDA-MB-231 and MDA-MB-435S cells are elongated and mesenchymal in morphology, with many cells exhibiting an easily recognizable leading edge, whereas the MDA-MB-453 cells are rounded and epithelium-like and do not exhibit polarized structures in culture.

## Conclusions

In summary, our studies show that Tiam1 expression correlates with motility in human breast cancer cell lines and is required to support the motile phenotype. Moreover, we demonstrated that endogenous Tiam1 is localized to the Golgi apparatus in breast tumor cells, where it regulates at least one Golgi function that is required to support motility. Because Tiam1 supports motility in breast tumor cells without any substantial effect on total cellular Rac-GTP levels, it may represent an attractive target for therapeutic intervention.

## Abbreviations

APC: adenomatous polyposis coli; BFA: Brefeldin A; DAPI: 4'6'-diamidine-2-phenylindole; DMEM: Dulbecco's Modified Eagle Medium; FBS: fetal bovine serum; GEF: guanine nucleotide exchange factor; HA: hemagglutinin; PAK: p21 activated kinase; Par1: protease-activated receptor 1; PBS: phosphate-buffered saline; Rac: Ras-related C3 botulinum toxin substrate; siRNA: small interfering ribonucleic acid; Tiam1: T-cell lymphoma invasion and metastasis-inducing protein.

## Competing interests

The authors declare that they have no competing interests.

## Authors' contributions

HCA designed the study, contributed to all of the experiments, and drafted the manuscript. ZL and RC assisted in the design and execution of the transwell motility assays. IPW led the conception and design of the study and supervised the research project. All authors read and approved the final manuscript.

## Supplementary Material

Additional file 1 **Supplementary Figure 1. Relative motility of breast cancer cell lines**. The indicated cell lines were examined in transwell motility assays, as described in Materials and Methods. Data shown are the average of three independent experiments performed in triplicate wells. Click here for file

Additional file 2 **Supplementary Figure 2. Overexpression of full-length Tiam1 increases Rac activity in MDA-MB-435S cells**. MDA-MB-435S cells were transiently transfected with full-length (FL) Tiam1, an HA-epitope tagged, activated derivative of Rac1 (Rac61L), or cognate vector (Vector). Lysates were collected and examined with Western blot for expression of total Rac1, Rac61L (HA), and Tiam1, and with affinity precipitation assay for levels of activated Rac1 (Rac1 GTP). The expression of α-tubulin was also measured as a loading control, as indicated. Data shown are representative of three independent experiments. Click here for file

Additional file 3 **Supplementary Figure 3. Endogenous Rac1 does not significantly colocalize with a Golgi marker in breast cancer cells**. Cell lines were plated on collagen I (50 μg/ml)-coated coverslips overnight, and then fixed and stained for Rac1 (green), GM130 (red), and DAPI (blue). Click here for file
