# Supplementary figures and images for: Regulation of breast cancer cell motility by T-cell lymphoma invasion and metastasis-inducing protein
Source: Breast Cancer Res. 2010 Sep 6;12(5):R69. doi: 10.1186/bcr2637 (PMC3096956; doi:10.1186/bcr2637)

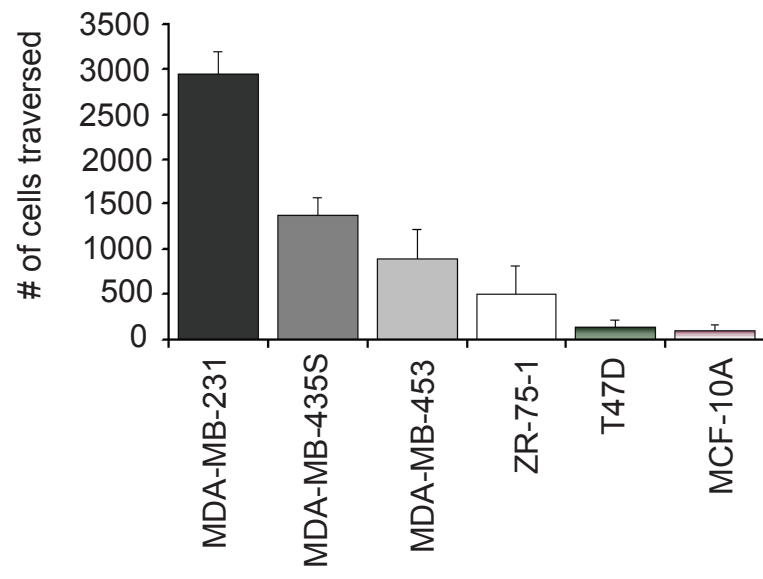

Supplement: Additional file 1 — Supplementary Figure 1. Relative motility of breast cancer cell lines. The indicated cell lines were examined in transwell motility assays, as described in Materials and Methods. Data shown are the average of three independent experiments performed in triplicate wells. [file bcr2637-S1.PDF]

Vector  
FL-Tiam1  
Rac61L-HA

Tiam1

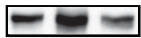

HA

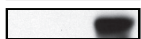

Rac1

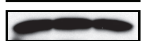

Rac1 GTP

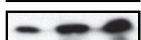

$\alpha$ -Tubulin

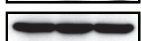

Supplement: Additional file 2 — Supplementary Figure 2. Overexpression of full-length Tiam1 increases Rac activity in MDA-MB-435S cells. MDA-MB-435S cells were transiently transfected with full-length (FL) Tiam1, an HA-epitope tagged, activated derivative of Rac1 (Rac61L), or cognate vector (Vector). Lysates were collected and examined with Western blot for expression of total Rac1, Rac61L (HA), and Tiam1, and with affinity precipitation assay for levels of activated Rac1 (Rac1 GTP). The expression of α-tubulin was also measured as a loading control, as indicated. Data shown are representative of three independent experiments. [file bcr2637-S2.PDF]

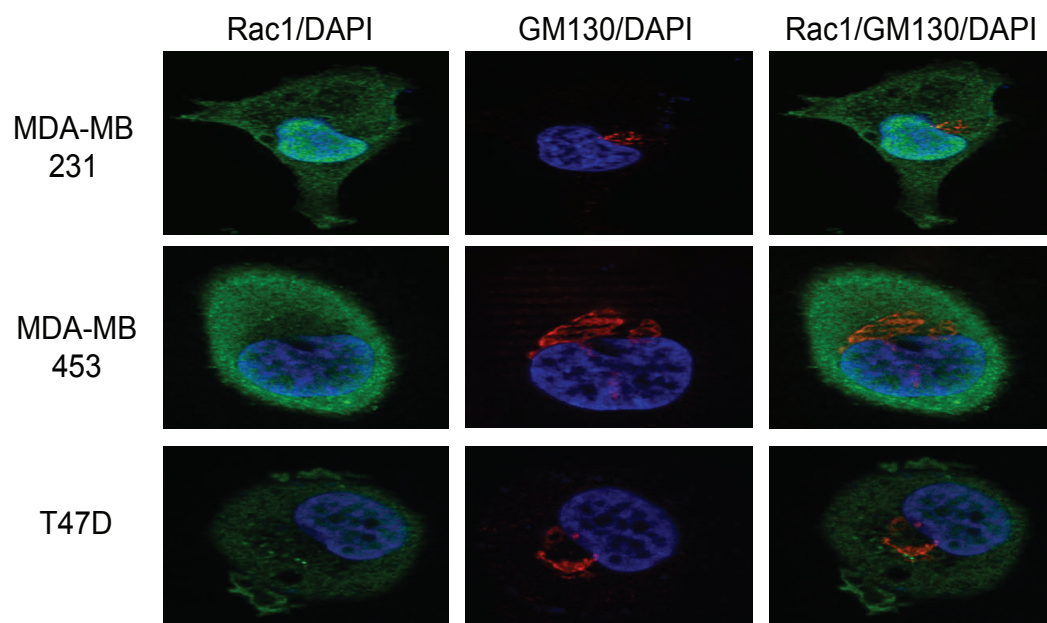

Supplement: Additional file 3 — Supplementary Figure 3. Endogenous Rac1 does not significantly colocalize with a Golgi marker in breast cancer cells. Cell lines were plated on collagen I (50 μg/ml)-coated coverslips overnight, and then fixed and stained for Rac1 (green), GM130 (red), and DAPI (blue). [file bcr2637-S3.PDF]
